# Selective Alleviation of Mitomycin C Sensitivity in *lexA3* Strains of *Escherichia coli* Demands Allele Specificity of *rif-nal* Mutations: A Pivotal Role for *rpoB87-gyrA87* Mutations

**DOI:** 10.1371/journal.pone.0087702

**Published:** 2014-02-03

**Authors:** Vinod Shanmughapriya, Shanmugaraja Meenakshi, M. Hussain Munavar

**Affiliations:** Department of Molecular Biology, School of Biological Sciences, Madurai Kamaraj University, Madurai, India; The University of Texas Health Science Center (UTHSCSA ), United States of America

## Abstract

Very recently, we have reported about an unconventional mode of elicitation of Mitomycin C (MMC) specific resistance in *lexA3* (SOS repair deficient) mutants due to a combination of Rif-Nal mutations (*rpoB87-gyrA87*). We have clearly shown that UvrB is mandatory for this unconventional MMC resistance in *rpoB87-gyrA87-lexA3* strains and *uvrB* is expressed more even without DNA damage induction from its LexA dependent promoter despite the uncleavable LexA3 repressor. The *rpoB87* allele is same as the *rpoB3595* which is known to give rise to a fast moving RNA Polymerase and *gyrA87* is a hitherto unreported Nal^R^ allele. Thus, it is proposed that the RNA Polymerase with higher elongation rate with the mutant DNA Gyrase is able to overcome the repressional hurdle posed by LexA3 to express *uvrB*. In this study we have systematically analysed the effect of three other *rpoB* (*rif*) mutations-two known to give rise to fast moving RNAP (*rpoB2* and *rpoB111*) and one to a slow moving RNAP (*rpoB8*) and four different alleles of *gyrA* Nal^R^ mutations (*gyrA199, gyrA247, gyrA250, gyrA259*) isolated spontaneously, on elicitation of MMC resistance in *lexA3* strains. Our results indicate that in order to acquire resistance to 0.5 µg/ml MMC cells require both *rpoB87* and *gyrA87* but resistance to 0.25 µg/ml of MMC can be brought about by either *rpoB87, gyrA87*, fast moving *rpoB* mutations or other *nal* mutations also. We have also depicted increased constitutive *uvrB* expression in strains carrying fast moving RNAP (*rpoB2* and *rpoB111*) with *gyrA87* and another *nal* mutation with *rpoB87* and expression level in these strains is lesser than *rpoB87-gyrA87* strain. These results evidently suggest an allele specific role for the *rif-nal* mutations to acquire MMC resistance in *lexA3* strains via increased constitutive *uvrB* expression and a pivotal role for *rpoB87-gyrA87* combination to elicit higher levels of resistance.

## Introduction

Mitomycin C (MMC) is a DNA intercalating agent that gives rise to DNA interstrand crosslinks. MMC has been used as an antitumor antibiotic that is naturally derived from *Streptomyces caespitosus* [1]. The DNA crosslinking by MMC leads to the induction of the SOS response in *Escherichia coli* by activating the RecA protein to its activated form called as RecA* and subsequent cleavage of the LexA repressor [2], [3]. The LexA is the SOS regulon repressor controlling the expression of more than 50 genes including most of the genes involved in repair of damaged DNA occurring due to exposure of cells to a wide range of DNA damaging agents [2], [4]. A mutant form of the repressor due to the mutation *lexA3*, renders the repressor nonreactive to the activated RecA* and thus fails to cleave itself. This mutation makes *E. coli* strains sensitive to all DNA damaging agents including MMC as the expression of all the SOS induced genes are turned off in *lexA3* strains [5]. But an alternate path for MMC repair in SOS deficient strains (*lexA3* or *recA*) was identified by Kumaresan and Jayaraman in 1988 and was termed as “SOS Independent Repair” or SIR. This SIR effect increased the MMC resistance of a *lexA3* strain by two additional spontaneously acquired mutations – A Rif resistant mutation, named *rpoB87,* mapped to the *rpoB* gene that codes for the β subunit of RNA Polymerase and a Nal resistant mutation, named *gyrA87,* mapped to *gyrA* gene that codes for the DNA Gyrase A subunit [6]. Our recent study on this unconventional mode of elicitation of Mitomycin C (MMC) resistance revealed an increase in *uvrB* expression even without DNA damage. This constitutive increase in *uvrB* expression was seen to be from its LexA dependent promoter despite the presence of uncleavable LexA3 repressor. It was shown that mutations inactivating the *uvrB* gene abolished this resistance. The study revealed that the *rpoB87* mutation carries a C_1565_→T_1565_ transition in the 522^nd^ codon of *rpoB* [7]. This lesion is the same as reported to be present in *rpoB3595* mutant as reported by Jin and Gross [8]. RNAP with RpoB3595 β subunit was shown to have increased termination read through/increased elongation rates [9], [10]. This allele is known to define a fast moving RNA Polymerase. The *gyrA87* is a hitherto unreported Nal^R^ allele that carries a G_244_→A_244_ transition changing the 82^nd^ codon of *gyrA* gene giving rise to a D82N change in the GyrA protein [7]. This defines a novel allele for Nalidixic acid resistance. An allele of *gyrA* giving a D82G mutation, when present alone, has been shown to exhibit low level of resistance to quinolone drugs but not to nalidixic acid [11]. This D82G mutation although not resistant to nalidixic acid like D82N mutation of *gyrA87*, has been shown to exhibit 2–3 fold decreased supercoiling activity *in vitro* [12] and also alters the steady state transcriptional activity of more than 800 genes [13]. Thus it is possible that the D82N mutant of DNA Gyrase A subunit also possesses altered negative supercoiling and a combination of the fast moving RNA Polymerase coupled with altered negative supercoiling can even increase the transcription of genes posing repressional roadblocks to the transcribing RNAP.

In this study, we have analysed whether the fast moving RNAP coded by the *rpoB87* Rif^R^ allele and the possible altered supercoiling due to mutant Gyrase coded by the *gyrA87* Nal^R^ allele only can play a role in the elicitation of MMC resistance as seen in phenotypically SIR^+^ strains or any other *rpoB* or *gyrA* allele(s) can also bring about the same effect. Thus, we have studied the effect of three other known Rif^R^ mutations, *rpoB2* (Fast moving RNAP), *rpoB8*(Slow moving RNAP) and *rpoB111* (Fast moving RNAP) [10], [14], [15] and four different alleles of spontaneously acquired Nal mutations on the MMC resistance of the SIR phenotype. The data reported here clearly imply allele specificity among the mutations affecting the β subunit of RNAP and mutations in the DNA gyrase A subunit in elicitation of MMC resistance of *lexA3* strains. We have also shown that this allele specific increase in MMC resistance of the *rpoB* and *gyrA* mutations is due to the activity of UvrB protein which arises as a result of difference in the basal level of *uvrB* expression in these strains.

## Materials and Methods

### Bacterial Strains Used

Given in Table 1 is the list of Bacterial strains used in this study. Genetic Nomenclature is according to Demerec *et al,* 1966 [16].

**Table 1:** List of *E. coli* K12 strains used in this study, their relevant Genotype and source.

Strain	Relevant Genotype	Source/Reference/Construction
AB1157	F^–^ *thr-1*, *araC14*, *leuB6*(Am),*Δ(gpt-proA)62*, *lacY1*, *supE44, hisG4*(Oc),*rpoS396*(Am), *rpsL31*(str^R^), *argE3*(Oc), *thi-1*	Laboratory collection
DM49	Same as AB1157 but *lexA3* Ind ^–^	M.K. Berlyn, CGSC, USA*
DM49N	Same as DM49 but *zfa*723::Tn*10 gyrA87*	Shanmughapriya and Munavar, 2012 [11]
DM49R	Same as DM49 but *argE^+^ rpoB87*	Shanmughapriya and Munavar, 2012 [11]
DM49RN	Same as DM49N but *argE^+^ rpoB87*	Shanmughapriya and Munavar, 2012 [11]
MMR1	F^–^Δ*lon510 cpsB10::lac, rpoB2*	Meenakshi and Munavar (Manuscript under preparation)
HR318	F-, *λ^−^*, *rph-1, btuB::*Tn*10*, *rpoB8*	Dr. Harinarayanan, CDFD, India^#^
NAM1	F-, *λ^−^*, *rph-1, rpoB111*	Agarwal, Shanmughapriya and Munavar (Unpublished work)
DM49R2N	Same as DM49N but *argE^+^ rpoB2*	This study, DM49N X P1/(MMR1)
DM49R8N	Same as DM49N but *argE^+^ rpoB8*	This study, DM49N X P1/(HR318)
DM49R111N	Same as DM49N but *argE^+^ rpoB111*	This study, DM49N X P1/(NAM1)
DM49RN3	Same as DM49R but *zfa*723::Tn*10 gyrA199*	This study
DM49RN4	Same as DM49R but *zfa*723::Tn*10 gyrA250*	This study
DM49RN7	Same as DM49R but *zfa*723::Tn*10 gyrA259*	This study
DM49RN9	Same as DM49R but *zfa*723::Tn*10 gyrA247*	This study
DM49R2	Same as DM49 but *argE^+^ rpoB2*	This study, DM49 X P1/(MMR1)
DM49R8	Same as DM49 but *argE^+^ rpoB8*	This study, DM49 X P1/(HR318)
DM49R111	Same as DM49 but *argE^+^ rpoB111*	This study, DM49 X P1/(NAM1)
DM49N3	Same as DM49 but *zfa*723::Tn*10 gyrA199*	This study, DM49 X P1/(DM49RN3)
DM49N4	Same as DM49 but *zfa*723::Tn*10 gyrA250*	This study, DM49 X P1/(DM49RN4)
DM49N7	Same as DM49 but *zfa*723::Tn*10 gyrA259*	This study, DM49 X P1/(DM49RN7)
DM49N9	Same as DM49 but *zfa*723::Tn*10 gyrA247*	This study, DM49 X P1/(DM49RN9)
JW0762-2	F*^−^*, Δ*(araD-araB)567, ΔlacZ4787(::rrnB-3*),Δ*uvrB751::kan*	M.K. Berlyn, CGSC, USA*
49RNUB	Same as DM49RN but Δ*uvrB751::kan*	Shanmughapriya and Munavar, 2012 [11]
49R2NUB	Same as DM49R2N but Δ*uvrB751::kan*	This study, DM49R2N X P1/(49RNUB)
49R8NUB	Same as DM49R8N but Δ*uvrB751::kan*	This study, DM49R8N X P1/(49RNUB)
49R111NUB	Same as DM49R111N but Δ*uvrB751::kan*	This study, DM49R111N X P1/(49RNUB)
49RN3UB	Same as DM49RN3 but Δ*uvrB751::kan*	This study, DM49RN3 X P1/(49RNUB)
49RN4UB	Same as DM49RN4 but Δ*uvrB751::kan*	This study, DM49RN4 X P1/(49RNUB)
49RN7UB	Same as DM49RN7 but Δ*uvrB751::kan*	This study, DM49RN7 X P1/(49RNUB)
49RN9UB	Same as DM49RN9 but Δ*uvrB751::kan*	This study, DM49RN9 X P1/(49RNUB)
JW0429-1	F ^–^, *Δ(araD-araB)567*, *ΔlacZ4787(::rrnB-3*),*Δlon-725::kan*, *λ^−^*, *rph-1*, *Δ(rhaD-* *rhaB)568*, *hsdR514*	M.K. Berlyn, CGSC, USA*
SM49LK	Same as DM49RN but Δ*lon::kan*	This study, DM49RN X P1/(JW0429-1)
SMM57LK	Same as AB1157 but Δ*lon::kan*	This study, AB1157 X P1/(SM49LK)
SMM49LK	Same as DM49 but Δ*lon::kan*	This study, DM49 X P1/(SM49LK)
SMM2LK	Same as DM49R2N but Δ*lon::kan*	This study, DM49R2N X P1/(SM49LK)
SMM8LK	Same as DM49R8N but Δ*lon::kan*	This study, DM49R8N X P1/(SM49LK)
SMM111LK	Same as DM49R111N but Δ*lon::kan*	This study, DM49R111N X P1/(SM49LK)
SMM3LK	Same as DM49RN3 but Δ*lon::kan*	This study, DM49RN3 X P1/(SM49LK)

* CGSC -Coli Genetic Stock Centre, USA.

**#** CDFD – Centre for DNA Fingerprinting and Diagnostics.

### Media and Chemicals

LB and minimal media [17] with appropriate supplements were used. Cells were routinely grown in LB at 37°C unless specified otherwise. Whenever required the following chemicals/antibiotics were added to the media in the final concentrations indicated. MMC (0.5 µg/ml or 0.25 µg/ml), Rif (20 µg/ml), Nal (20 µg/ml), Tet (10 µg/ml), Kan (45 µg/ml), Amino acids (30 µg/ml). The chemicals used were purchased from Himedia, India, Sigma, USA, Qiagen, India, Invitrogen, India and Sisco laboratories, India. MMC was purchased from Biobasic. Inc. India. The primers used for the study were obtained from Chromous Biotech, Bangalore, India. The enzymes used were obtained from Fermentas, India.

### Mobilization of *rpoB2, rpoB8* and *rpoB111* Mutations into DM49N (*gyrA87-lexA3*) and DM49 (*lexA3*) Strains

All P1 mediated transductions were performed as described in Miller [17], [18]. *argE^+^*and *rpoB* are linked and therefore cotransduce. Hence, using the P1 lysate made directly from the strains carrying the various *rpoB* mutations, the *argE^+^* marker was transduced into *argE* ^–^ recepients, DM49N and DM49 (Table 1). The Arg^+^ transductants obtained on selective minimal plates lacking Arginine were screened for Rif resistant colonies.

### Isolation, Mapping and Sequence Analyses of *gyrA* Nal^R^ Mutants from DM49R (*rpoB87-lexA3*) Strain

Spontaneous Nalidixic acid resistant mutants were isolated by plating 100 µl of overnight cultures of the DM49R strain in LB plates containing 20 µg/ml of Nalidixic acid. 12 such Nal^R^ mutants of DM49R strain were isolated in four independent set of experiments. The obtained Nal^R^ mutants were then mapped to the *gyrA* gene initially by transducing *zfa*723::Tn*10* linked to *gyrA* to obtain Tet^R^ mutants and then looking for the cotransduction of Nal^R^ phenotype with *zfa*723::Tn*10*. Subsequently, the 546 bp region spanning the Nal resistance region of all the 12 *gyrA* mutants were amplified using the the forward primer nalF : ATGAGCGACCTTGCGAGAGA and the reverse primer nalR : CGGGATGTTGGTTGCCATAC. The obtained products were checked on 1% agarose gels along with a 1kb DNA ladder and purified using DNA extraction Kit obtained from Fermentas, India. The purified PCR products were then sequenced by Chromous Biotech, Bangalore, India. The sequence results obtained were then analysed with BLAST tool in NCBI nucleotide database and mutations/mismatches in DNA sequence of *E. coli* K12 substrain MG1655 obtained manually analysed.

### Mitomycin C Survival Analyses

Overnight cultures were sub-cultured into fresh LB broth and grown for ∼8–9 hours. The samples were then diluted in fresh 0.85% saline solution and appropriate dilutions were spotted on LB plates and on LB plates containing relevant concentration of MMC for the cell titre. The % survival of the strains was calculated when required with the cfu/ml on MMC plates and the cfu/ml on LB plates.
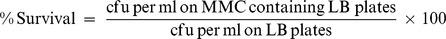



In other cases the plates were scanned and the growth on MMC plates were analysed qualitatively and the plates were photographed. The growth on LB plates was analysed ∼12–14 hours after the spotting while the growth on MMC containing plates were analysed after ∼24 hours of incubation.

### P1 Transduction Mediated Construction of *uvrB::kan* and *lon::kan* Markers into the Relevant Strains

P1 lysate prepared from the relevant donor strains for each cross (Table 1) was used to transfer the relevant marker (Kanamycin resistance) into recipient strains and the transduced cells were then plated on appropriate selective plates containing Kanamycin. The plates were incubated at 37°C until transductants appeared. The obtained transductants were segregated by streaking them on appropriate antibiotic containing LB agar plates. Representative transductants in each case were purified and stored for further analyses. The relevant recipient and donor strains are also mentioned in Table 1.

### RT-PCR Analyses

Reverse Transcription PCR was performed by isolating RNA from late-log phase cultures of the respective strains using the RNA isolation kit from Qiagen, India. The RNA obtained was then normalized with corresponding OD at 260nm and used as template for Reverse Transcription. First step cDNA was obtained from the RNA samples using the cDNA synthesis kit from Invitrogen, India. The PCR with first strand cDNA for *uvrB* was performed with uvrBRTFor 5′-CGACGCTGTTTGATTACCTG-3′, uvrBRTRev 5′-CTACCAGCACGTCGAACTCA-3′. The PCR reaction was done with thirty amplification cycles with the following conditions. 15 min at 94°C, 30 sec at 94°C, 45 sec at 55°C, 30 sec at 72°C, 7 min at 72°C and maintained at 4°C. The amplicon size was verified by agarose gel electrophoresis. A PCR reaction with DNA as sample was used as a positive control reaction and one with RNA as sample was used as negative control to eliminate possibility of DNA contamination in RNA sample. Equal volumes of RT-PCR samples were loaded in each lane to get to know the *uvrB* expression level.

### Filamentation Analyses

Relevant strains were grown till mid log phase under Mitomycin C induced and un-induced conditions. The un-induced cultures were directly heat fixed on glass slides, stained with saffranin red and viewed. For inductions, the mid-log phase cells were treated with 0.5 µg/ml of MMC for 2 hours before processing and about 10 µl of each culture was spotted, smeared and the samples were directly heat fixed on glass slides, stained with saffranin red and viewed. The results were then analysed by observing the stained preparations of the cells under light microscope with 40x resolution. More than 10 frames of the slides were then observed under the Nikon Eclipse Ti light microscope at 40x resolution and images were viewed and photographed using the software NIS elements D version 3.0.

## Results

### Effect of *rpoB2, rpoB8* and *rpoB111* with *gyrA87* on MMC Resistance of *lexA3* Strains

**Figure 1:**
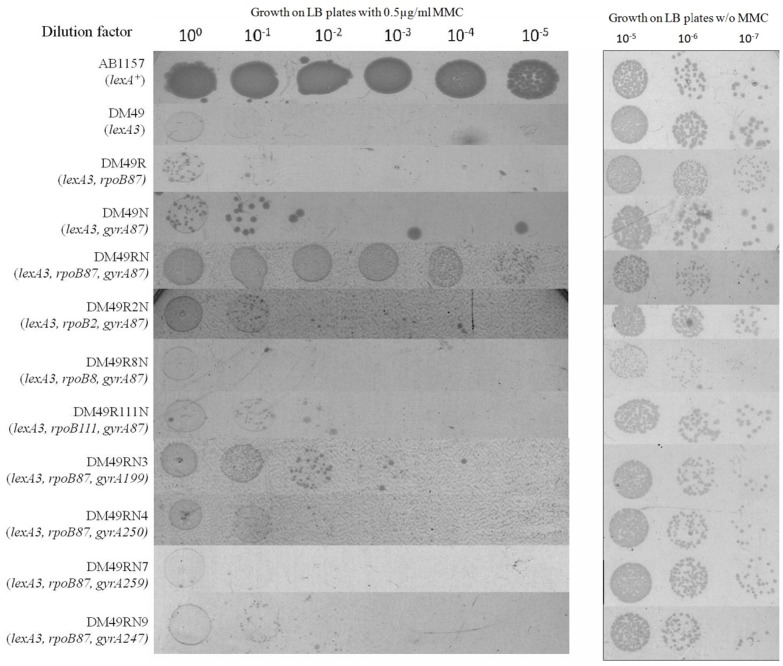
Sequential spotting test for analyses of survival of various Rif-Nal strains on LB plates containing 0.5 µg/ml of MMC after ∼24 hours incubation. Appropriate control strains (AB1157 for positive control and DM49 for Negative control) were also tested. The growth of the respective strains on LB plates without MMC after ∼12–14 hours incubation is given on the right.

**Figure 2:**
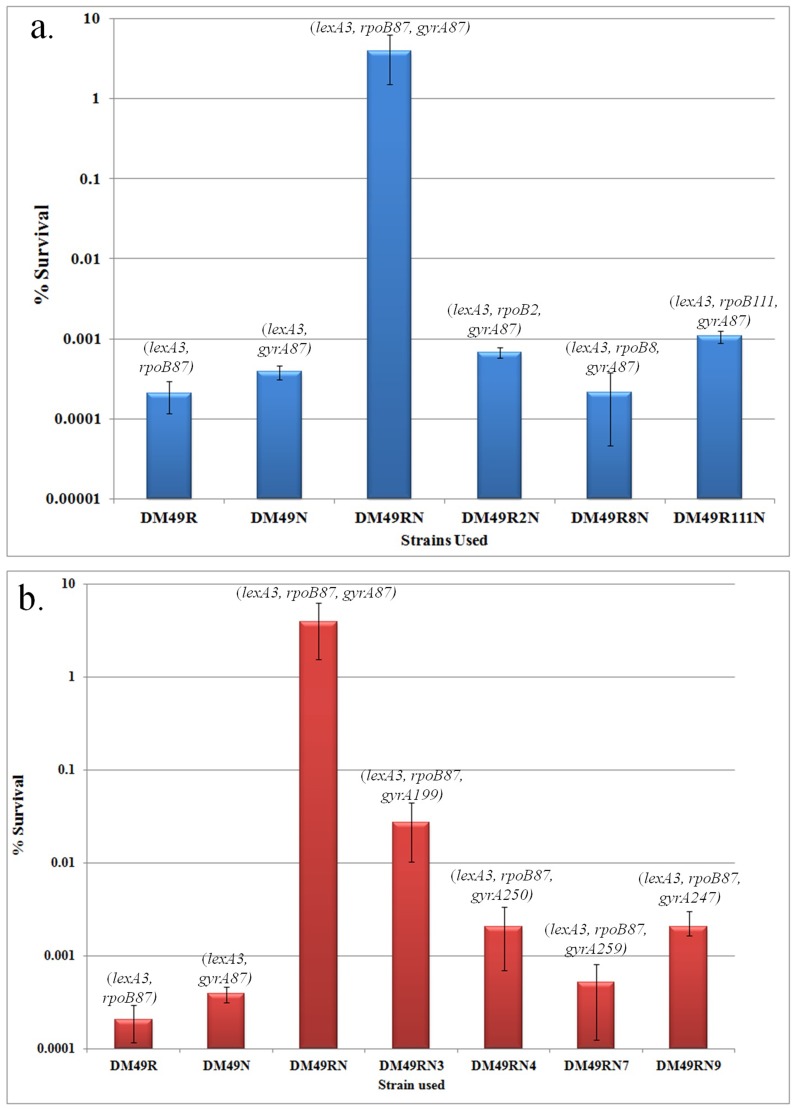
Survival of different *rif-nal* strains on MMC plates. a. % survival of relevant *gyrA87-*Rif^R^ strains on LB plates containing 0.5 µg/ml of MMC. b. % survival of relevant *rpoB87-*Nal^R^ strains on LB plates containing 0.5 µg/ml of MMC. The values plotted are the average of three independent set of experiments. The standard error values are given as error bars. The % survival values were calculated as (cfu per ml in LB with MMC plates/cfu per ml in LB plates w/o MMC) X100.

Three Rifampicin resistant mutations other than the *rpoB87*/*rpoB3595* mutation were used to study their effect on elicitation of SIR phenotype. Two of these three mutations (*rpoB2* and *rpoB111*) are known to give rise to fast moving RNA Polymerases and one mutation *rpoB8*, was known to give rise to a slow moving RNA Polymerase as mentioned in the introduction section. The *rif* alleles, base changes, amino acid changes and known relevant phenotypes of all the *rpoB* mutations are listed in Table 2. The *rpoB2, rpoB8* and *rpoB111* mutations were introduced into DM49N (*lexA3 gyrA87*) strain as mentioned in materials and methods. The relative survival of various strains was carried out at 37°C in this study as opposed to the 30°C assays used in the previously published data of MMC survival [7] because during the course of this investigation we found that the resistance to MMC was found to be a little better at higher temperatures compared to 30°C (Shanmughapriya and Munavar, Unpublished data). MMC survival analyses of the relevant strains clearly indicate that none of the other mutations could make the *lexA3* strain to survive on 0.5 µg/ml MMC containing plates but the *rpoB87-gyrA87*-*lexA3* strain could survive well at the same concentration (Fig. 1). Quantification of the MMC survival of the strains carrying respective *rif* mutation revealed that the DM49R2N and DM49R111N carrying the two fast moving RNAP alleles *rpoB2* and *rpoB111* respectively show a marginal (<5 fold) increase in MMC resistance which was not observed in the DM49R8N with the slow moving RNAP allele *rpoB8* (Fig. 2a). But even this resistance with fast moving RNAP alleles was still >1000 fold less than that observed with the DM49RN strain bearing *rpoB87-gyrA87* alleles (Fig. 2a). The survival of all the strains on LB plates was found to be in the order of ∼10^9^ cfu/ml.

**Table 2:** *rpoB* alleles used in this study and their relevant characteristics.

*rpoB* allele	Base pair change/Codonor amino acid change	Type of RNAPproduced	Relative Termination read through*		Source/Comments
			Rho independent terminators	Rho dependent terminators	
*rpoB87/rpoB3595*	C_1565_ →T_1565_/TCT→TTT(S522F)	Fast moving	2.5–3.5	2–2.7	Kumaresan and Jayaraman, 1988, Shanmughapriya and Munavar, 2012, Jin and Gross, 1988
*rpoB2*	C_1576_ →T_1576/_CAC→TAC(H526Y)	Fast moving	1.3–3.3	1.1–1.6	Meenakshi and Munavar, Unpublished data
*rpoB8*	A_1538_→C_1538/_CAG→CCG(Q513P)	Slow moving	0.3–0.6	0.4–0.8	R. Harinarayanan, CDFD, Hyderabad, India
*rpoB111*	C_1692_→T_1692_/CCT→CTT(P564L)	Fast moving	1.5–2.5	1.1–1.4	This study

* Values given as fold increase in termination read through are from Jin *et al*, 1988.

### Effect of Spontaneous Nal^R^ Mutants with *rpoB87* on MMC Resistance of *lexA3* Strains

12 spontaneous Nalidixic acid resistant mutants were isolated and were mapped to the *gyrA* gene as described in materials and methods section. Sequencing of the Nal^R^ region of these alleles indicated that the 12 mutations defined four different alleles of *gyrA* gene and these alleles were named *gyrA199, gyrA250, gyrA259* and *gyrA247*. Although these *nal* alleles have been previously reported by different groups in various contexts, this is the first study where their roles have been analysed in relation with DNA repair. The *gyrA* allele, base change and codon/amino acid change in each of these four different Nal^R^ mutants are listed in Table 3. The MMC resistance of the strains DM49RN3, DM49RN4, DM49RN7 and DM49RN9 carrying each of the four different alleles together with *rpoB87* was then studied. Survival of these strains on LB plates containing 0.5 µg/ml of MMC indicates that none of these strains could survive as efficiently as like the DM49RN strain carrying the *rpoB87-gyrA87* combination (Fig. 1). Quantification of this survival indicated that although the *gyrA* mutants when present along with *rpoB87* increased the resistance of the strains up to 50 fold, this resistance was still >50–100 fold less than that observed in with *rpoB87-gyrA87-lexA3* strain DM49RN (Fig. 2b).

**Table 3:** Sequence changes in various *gyrA* alleles used in the study.

*gyrA* allele	Base pair change/Codon and amino acid change	Source/comments
*gyrA87*	G_244_→A_244/_GAC→AAC (D82N)	Kumaresan and Jayaraman, 1988, Shanmughapriya and Munavar, 2012
*gyrA199*	G_199_→T_199_/GCC→TCC (A67S)	Isolated and sequenced in this study
*gyrA250*	G_250_→T_250_/GCG→TCG (A84S)	Isolated and sequenced in this study
*gyrA259*	G_259_→A_259_/GAC→AAC (D87N)	Isolated and Sequenced in this study
*gyrA247*	T_247_→G_247/_TCG→GCG (S83A)	Isolated and Sequenced in this study

### Reduced ‘SIR’ Effect due to *rif* and *nal* Alleles Exclusive of the Other

**Figure 3:**
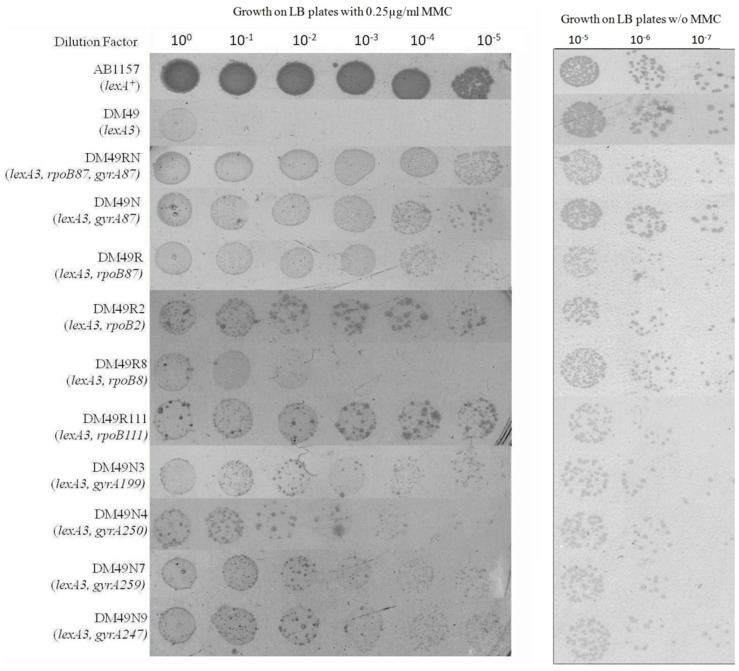
Sequential spotting test for analyses of survival of various Rif-Nal strains on LB plates containing 0.25 µg/ml of MMC after ∼24 hours incubation. Appropriate control strains (AB1157 for positive control and DM49 for Negative control were also tested). The growth of the respective strains on LB plates without MMC after ∼12–14 hours incubation is given on the right.

While the level of resistance to 0.5 µg/ml of MMC observed in *rpoB87-gyrA87*-*lexA3* strains could not be matched with resistance level of any other *rif/nal* allelic combination, it is still tenable that some of these other mutations can display increased resistance to lower concentrations of MMC in combination or in isolation. Thus, the survival of these strains was checked on LB plates containing 0.25 µg/ml of MMC. At this decreased concentration of MMC while the *lexA3* strain DM49 was still unable to grow, the *rpoB87-lexA3* strain DM49R and the *gyrA87-lexA3* strain DM49N also grew well foregoing the need for both the mutations to be present. This effect can be called the reduced ‘SIR’ effect and either the *rpoB87* or *gyrA87* mutation alone could elicit this effect. Therefore, other Rif^R^-*gyrA87-lexA3* strains and the Nal^R^-*rpoB87-lexA3* strains were also growing well on 0.25 µg/ml MMC. Thus, the relevant *rif* or *nal* alleles were alone transduced into the *lexA3* strain (DM49) without the *gyrA87* or *rpoB87* allele (as described in Materials and methods) to analyse their reduced ‘SIR’ effect. It was seen that the *rpoB2* and *rpoB111* alleles are able to alleviate the MMC sensitivity of the DM49 strain at 0.25 µg/ml MMC (Fig. 3). The *gyrA199, gyrA250, gyrA259* and the *gyrA247* alleles were also able to increase the resistance of the DM49 strain. But this reduced ‘SIR’ effect was not seen only with the *rpoB8* slow moving RNAP allele (Fig. 3).

### Pivotal Role for UvrB in Increased MMC Resistance Seen in Strains with Different *rif-nal* Alleles Tested

As could be seen from the above results, it is evident that there exists some level of allele specificity among the *rif* and *nal* mutations in eliciting resistance to even low level of MMC. It was shown in our previous study that the increased MMC resistance of the *rpoB87-gyrA87-lexA3* strain was due to the increase in constitutive/basal level of expression of the gene *uvrB.* Other studies have shown that out of all the Nucleotide Excision Repair functions (UvrA, UvrB and UvrC/Cho) that take part in repair of MMC induced DNA damage only UvrB is mandatory [19]. Our results which showed that increase in expression of the *uvrB* gene in a *lexA3* strain could increase the MMC resistance correlated well with the above observation [7]. This places UvrB in a prominent role in SIR mediated MMC resistance observed in SOS deficient strains. Thus, it is quite possible that it is the increased expression/activity of UvrB that is also causing the little increase in MMC resistance observed in the other *rif-nal* alleles tested in this study.

Therefore, we ventured to study the effect of inactivation of the *uvrB* gene on the MMC resistance of the *lexA3* strains carrying the different *rif-nal* allelic combinations. For this purpose, we introduced the Δ*uvrB::kan* allele from relevant donor strain (see Table 1) into all the *rif-nal-lexA3* strains analysed in this study. This was done using P1 mediated transduction as given in the materials and methods section. The relevant genotypes of the *uvrB* inactivated derivatives of all the *rif-nal-lexA3* strains analysed in this study are given in Table 1. The growth of the relevant Δ*uvrB::kan* derivatives along with respective parental controls were checked on LB plates containing 0.5 µg/ml MMC. It was seen that inactivation of *uvrB* gene in all of the *rif-nal-lexA3* strains led to loss of the very little MMC resistance (if any) observed in the strain. The resistance of these mutants to 0.25 µg/ml MMC was also checked and it was observed that the *uvrB* inactivated mutants were also totally sensitive to 0.25 µg/ml MMC. It was observed that the Δ*uvrB::kan lexA^+^* derivative of parent strain, AB1157 was not completely sensitive to 0.25 µg/ml MMC but displayed a very very sick growth. However, this strain was completely sensitive to 0.5 µg/ml MMC. This may be possibly due to the fact that in AB1157 all the other SOS repair functions are fully active and thus might be capable of giving rise to a much diminished survival to very low concentrations of MMC. The phenotypic effects of the relevant strains on survival to MMC is summarised in Table 4. From this observation, it is clear that the increased MMC resistance of the *gyrA87-lexA3* strains with *rpoB2* or *rpoB111* is indeed a result of *uvrB* gene function. Likewise, in an *rpoB87-lexA3* strain carrying *gyrA199, gyrA250, gyrA259* or *gyrA247,* increased MMC resistance is due to the function of UvrB.

**Table 4:** Level of MMC survival in relevant strains and its implications.

Strain	Relevant Genotype	MMC survival at0.5 µg/ml/(R/S)	MMC survival at0.25 µg/ml/(R/S)	Possible explanation/Reasons/comments
AB1157	*lexA* ^+^ *rpoB^+^ gyrA^+^*	R (+++)	R (+++)	Completely resistant toDNA damaging agents being *lexA^+^*
DM49	*lexA3 rpoB^+^ gyrA^+^*	S (−)	S (−)	Sensitive to all DNA damagingagents being *lexA3*, SOS uninducible strain
DM49RN	*lexA3 rpoB87 gyrA87*	R (++)	R (++)	Selective suppression of onlyMitomycin C sensitive phenotype of*lexA3* by *rpoB87 gyrA87* mutations dueto expression of *uvrB.*
DM49R	*lexA3 rpoB87 gyrA^+^*	S (−)	R(++)	Reduced SIR effect due to only *rpoB87*fast moving RNAP producing allele
DM49N	*lexA3 gyrA87 rpoB^+^*	S (−)	R(++)	Reduced SIR effect due to only *gyrA87*
DM49R2N	*lexA3 rpoB2 gyrA87*	S (−)	R (++)	Reduced SIR effect due to only*gyrA87* and perhaps *rpoB2*
DM49R8N	*lexA3 rpoB8 gyrA87*	S (–)	R (++)	Reduced SIR effect due to only *gyrA87*
DM49R111N	*lexA3 rpoB111 gyrA87*	S (−)	R (++)	Reduced SIR effect due to only*gyrA87* and perhaps *rpoB111*
DM49RN3	*lexA3 rpoB87 gyrA199*	S (−)	R (++)	Reduced SIR effect due to only*rpoB87* fast moving RNAP producing allele
DM49RN4	*lexA3 rpoB87 gyrA250*	S (−)	R (++)	Reduced SIR effect due to only*rpoB87* fast moving RNAP producing allele
DM49RN7	*lexA3 rpoB87 gyrA257*	S (−)	R (++)	Reduced SIR effect due to only*rpoB87* fast moving RNAP producing allele
DM49RN9	*lexA3 rpoB87 gyrA247*	S (−)	R (++)	Reduced SIR effect due to only*rpoB87* fast moving RNAP producing allele
49RNUB	*lexA3 rpoB87 gyrA87*Δ*uvrB::kan*	S (–)	S (–)	Complete loss of MMC resistancedue to loss of *uvrB* function
49R2NUB	*lexA3 rpoB2 gyrA87*Δ*uvrB::kan*	S (–)	S (–)	Complete loss of MMC resistance dueto loss of *uvrB* function
49R8NUB	*lexA3 rpoB8 gyrA87*Δ*uvrB::kan*	S (–)	S (–)	Complete loss of MMC resistance dueto loss of *uvrB* function
49R111NUB	*lexA3 rpoB111 gyrA87*Δ*uvrB::kan*	S (–)	S (–)	Complete loss of MMC resistancedue to loss of *uvrB* function
49RN3UB	*lexA3 rpoB87 gyrA199*Δ*uvrB::kan*	S (–)	S (–)	Complete loss of MMC resistancedue to loss of *uvrB* function
49RN4UB	*lexA3 rpoB87 gyrA250*Δ*uvrB::kan*	S (–)	S (–)	Complete loss of MMC resistance due to loss of *uvrB* function
49RN7UB	*lexA3 rpoB87 gyrA257*Δ*uvrB::kan*	S (–)	S (–)	Complete loss of MMC resistancedue to loss of *uvrB* function
49RN9UB	*lexA3 rpoB87 gyrA247*Δ*uvrB::kan*	S (–)	S (–)	Complete loss of MMC resistancedue to loss of *uvrB* function

R (+++) – ∼100% survival.

R (++) – ∼1% survival.

S (−) – ∼0.01–0.001% survival.

S (–) – ∼ 0.0001–0.00001% survival.

S (–) – Complete loss of survival.

### Differential Basal Level of *uvrB* Expression in the Different *rif-nal-lexA3* Combinations

**Figure 4:**
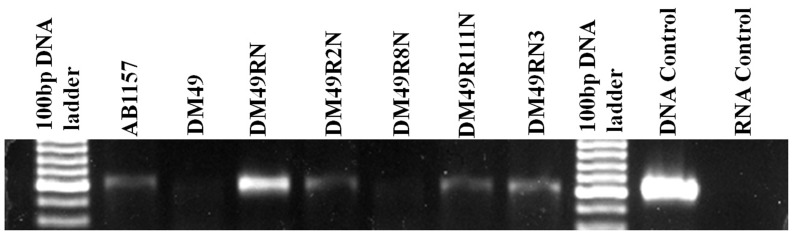
RT-PCR based analysis of expression of *uvrB* in indicated strains along with DNA control as positive reaction and RNA control as negative reaction.

It is evident from the above results that UvrB once again plays the central role in MMC resistance observed in the various *rif-nal-lexA3* strains like seen with *rpoB87-gyrA87-lexA3* strain. It was observed in our previous study that this increase in UvrB activity in *rpoB87-gyrA87-lexA3* strain stems from an increased *uvrB* gene expression. We thus analysed whether the role of UvrB in the other *rif-nal-lexA3* strains also corresponds with increase in the constitutive/basal level of expression of *uvrB* gene as was seen in the *rpoB87-gyrA87-lexA3* strain. Thus, semi-quantitative RT-PCR analyses were performed to analyse the level of *uvrB* transcript in several of these *rif-nal-lexA3* strains (DM49RN, DM49R2N, DM49R8N, DM49R111N and DM49RN3) along with relevant parental controls (AB1157 and DM49). As was expected, it was seen that the basal level of *uvrB* transcript was much higher in the DM49RN (*rpoB87, gyrA87, lexA3*) strain compared with that of the other strains (Fig. 4). It was also seen that the basal *uvrB* transcript level of the DM49 (*lexA3*) strain was decreased as compared to its *lexA* ^+^ parent strain AB1157. This may be perhaps due to the fact that LexA3 is an uninducible repressor and it might possibly possess decreased dissociation constant from its DNA binding region: A view which is conjectural. This may be the cause for the diminished transcript levels from DM49 compared to AB1157. Our results clearly indicate that DM49R2N (*rpoB2, gyrA87, lexA3*), DM49R111N (*rpoB111, gyrA87, lexA3*), DM49RN3 (*rpoB87, gyrA199, lexA3*) strains were slightly increased compared to the DM49 (*lexA3*) strain (Fig 4). It was also seen that the *uvrB* transcript levels of the DM49R8N strain carrying the slow moving RNAP is further decreased (Fig 4). This indicates that *uvrB* expression is central in giving increased resistance to MMC in a *lexA3* strain and that only a fast moving RNAP can bring about this effect. Also this shows that the elongation rates of the RNAP present in the strain plays a major role is increasing *uvrB* expression along with *gyrA87*.

### Analyses of Extent of Filamentation Observed in Δ*lon* Derivatives of Different *rif-nal-lexA3* Combinations

The hallmark of the SIR phenotype as seen from our previous results is the differential expression of genes in the SOS regulon in a *lexA3* strains. It has been shown that in the DM49RN strain, while the expression of *uvrB* gene is increased, the expression or activity of another SOS gene, *sulA*, is unaffected. Thus, the effect of *rpoB87* and *gyrA87* mutations seemed to be limited to expressing only one (or possibly few) of the SOS induced genes in a *lexA3* strain. Thus we believe that the same or similar mechanism is in action in elicitation of MMC resistance seen in the other *rif-nal* mutations where only *uvrB* is expressed but not *sulA.* We thus ventured to confirm whether the same is true in few other selected *rif-nal* allelic combinations in a *lexA3* strain. For this purpose we tested whether the activity of the SulA, cell division inhibitor, is increased in the strains carrying the fast moving RNAP producing allele with *gyrA87*. SulA has been shown to specifically bind the cell division protein FtsZ and stall cell division during DNA repair [20]. After repair, the SulA protein is degraded by the Lon protease and thus enables progression of the cell division. It has been known that in absence of Lon protease expression of *sulA* by DNA damage would lead to irreversible stalling of cell division and thus the cells will be seen to give rise to filamentation [21], [22]. Thus, the expression/activity of the SulA protein can be tested by checking the extent of filamentation of a strain lacking Lon protease.

**Figure 5:**
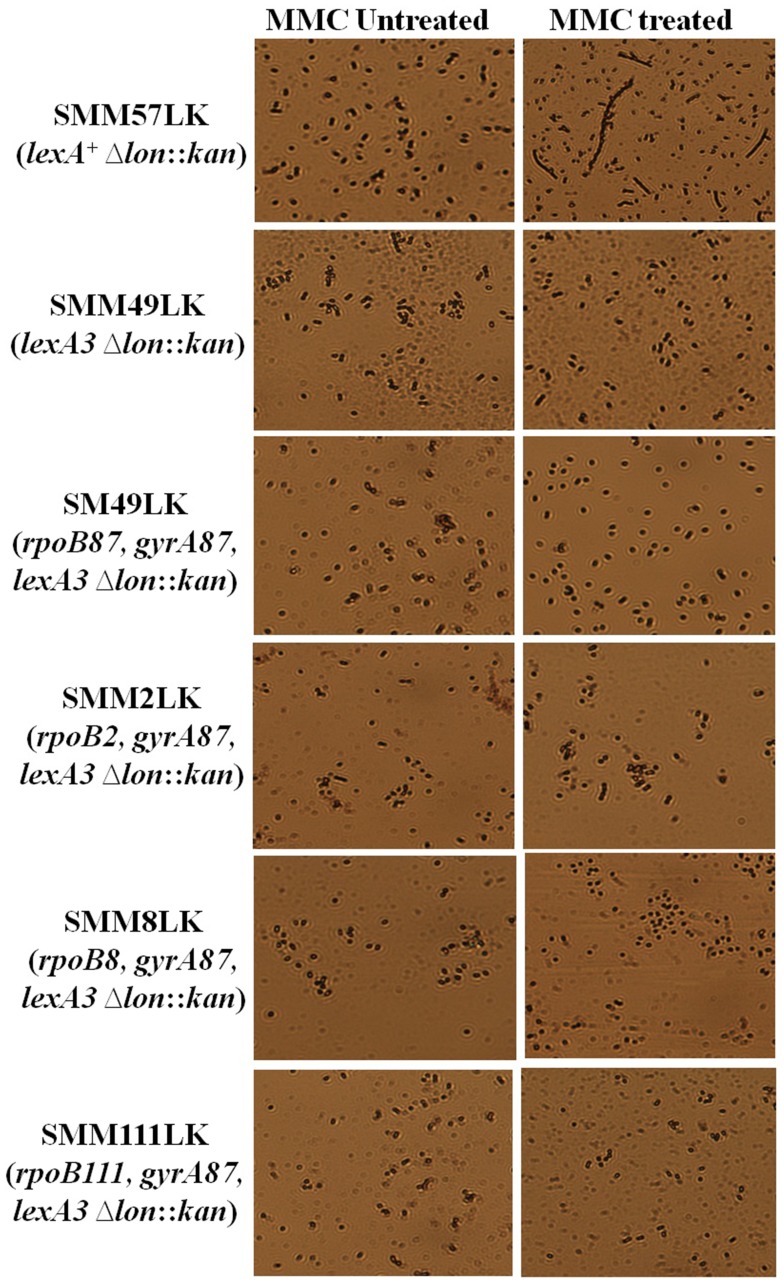
Extent of filamentation in MMC untreated and 0.5 µg/ml MMC treated samples of indicated strains as observed under light microscope. Relevant genotypes of the strains are mentioned wherever appropriate.

For this purpose, *lon* inactivated derivatives of all the *rif-gyrA87-lexA3* strains analysed in this study were constructed as described in materials and methods. The relevant genotype of the Δ*lon* derivatives of the strains are given in the Table 1. The filamentation levels of each of these derivatives were tested as given in materials and methods. It was seen from the results observed that there was no filamentation seen with any of the *rif-nal-lexA3* strains tested (Fig. 5). AB1157 was used as a positive control for observation of filamentation while DM49 was used as a negative control. This result shows that the effect of the fast moving *rif* and other *nal* alleles is very selective and increases only *uvrB* expression and they apparently do not affect the expression or activity of the *sulA* gene and its product as is the case with *rpoB87-gyrA87-lexA3* strain.

## Discussion

The process of transcription has been linked to DNA repair mechanisms for a long time now. This was termed as Transcription Coupled Repair which is a subpathway of the Nucleotide Excision Repair mechanisms involving the UvrABC proteins [23] and mutations in RNA Polymerase β subunit have been shown to be defective in transcription coupled DNA repair mechanisms [24], [25]. The role of these *rpoB* mutations in DNA repair were not directly affecting the expression of genes involved in repair mechanism but implicated in processing and removal of lesions. But *rpoB3595* mutation has been shown to be indirectly involved with the DNA repair processes. It was shown that the *rpoB3595* mutation increases the expression of SOS genes in a strain that is constitutive for SOS [26]. In our previous study, we have shown that this increase in expression of *uvrB* is seen even in *lexA3* strains when the *rpoB87/rpoB3595* mutation is coupled with the *gyrA87* mutation [7].

The results that we have reported herein pertaining to the MMC survival patterns of different Rifampicin and Nalidixic Acid resistant mutations clearly reveal that the SIR effect observed in *rpoB87* and *gyrA87* mutations are highly specific to those mutations. Survival of all the other *gyrA87* coupled Rif mutations other than *rpoB87* and *rpoB87* coupled Nal mutations other than *gyrA87* was highly impaired on continuous exposure to 0.5 µg/ml of MMC. When the concentration of MMC was decreased to 0.25 µg/ml other fast moving RNA Polymerases alone and some Nal resistant mutations also were able to resist it and give rise to survivors. Our results presented here clearly implicate the central role for UvrB in MMC resistance of *lexA3* strains carrying fast moving RNAP producing *rif* alleles with *gyrA87* and other *nal* alleles with *rpoB87*. It is also seen that this role of UvrB arises from the increased expression of basal level of *uvrB* in *gyrA87-lexA3* strains carrying any fast moving RNAP producing allele (*rpoB3595, rpoB2* and *rpoB111*) or in *rpoB87-lexA3* strain with *gyrA199*. Even among the strains with fast moving RNAP, *rpoB87/rpoB3595* gives rise to the highest *uvrB* expression while those carrying *rpoB2* and *rpoB111* gave a lesser increase in *uvrB* expression. It was seen that *rpoB87* with other Nal mutation *gyrA199* also gives a slight increase in *uvrB* expression as seen with *gyrA87* mutants with *rpoB2* and *rpoB111.* It is also evident that it is indeed the UvrB protein that causes the increased resistance with the other alternate Nal alleles tested because inactivation of the *uvrB* gene gives rise to loss of MMC resistance in all these strains. But, the *lexA3* strain carrying *rpoB87* mutation also needs only *gyrA87* to give rise to the highest *uvrB* expression and consequently the highest MMC resistance.

Global relaxation of DNA supercoiling has also been shown to have a great impact on gene expression and it has been shown that this can lead to decrease in expression of some genes while the expression of number of other genes is increased [27]. Also, as mentioned previously, the D82G mutation affecting the same position as that of the D82N mutation in *gyrA87,* was seen to affect the transcriptional levels of more than 800 genes [13]. Thus, the *rpoB87* and *gyrA87* mutations can be perceived to affect the expression of genes like *uvrB* when combined together. As we have reported here, the increased elongation rate of the RNAP along with the possible altered DNA Gyrase activity caused by *rpoB87* and *gyrA87* mutations could be one of the major reasons for this increased expression of *uvrB* in *lexA3* strains carrying the various *rif* and *nal* alleles. Although this increase in expression of *uvrB* gene can be attributed to the higher elongation rate of the RNAP produced, the effect need not be solely due to that. It may be even be specific to the *nal* allele present as our results given here indicate and probably needs the faster RNAP such as that from *rpoB87/rpoB3595* along with *gyrA87* mutation affecting specifically the codon 82 to give the best MMC resistance. The results pertaining to MMC resistance in spontaneously acquired mutations in *rpoB* and *gyrA* imply high relevance in the study of resistance to MMC in *E. coli* and may pave way for the better understanding of the mode of resistance of *E. coli* to MMC. Owing to the fact that we have shown clearly that an SOS function (*uvrB*) is needed for elicitation of SIR phenotype, we retained the same acronym SIR but referring to SOS Interdependent Repair instead of originally named SOS Independent Repair.
